# MRI-based adrenal gland volume is associated with cardiovascular alterations in individuals without prior cardiovascular disease

**DOI:** 10.1038/s41598-024-65673-2

**Published:** 2024-06-25

**Authors:** Esther Askani, Susanne Rospleszcz, Roberto Lorbeer, Charlotte Wintergerst, Katharina Müller-Peltzer, Johanna Nattenmüller, Dunja Hasic, Ricarda von Krüchten, Elias Kellner, Marco Reisert, Wolfgang Rathmann, Annette Peters, Christopher L. Schlett, Fabian Bamberg, Corinna Storz

**Affiliations:** 1 Department of Diagnostic and Interventional Radiology, Medical Center ‐ University of Freiburg, Faculty of Medicine, University of Freiburg, Freiburg, Germany; 2 Department of Epidemiology, Institute for Medical Information Processing, Biometry, and Epidemiology, Ludwig-Maximilians-University Munich, Munich, Germany; 3 Institute of Epidemiology, Helmholtz Centre Munich, German Research Center for Environmental Health, Neuherberg, Germany; 4 German Center for Cardiovascular Disease Research (DZHK E.V.), Munich, Germany; 5 Department of Radiology, Ludwig-Maximilans-University Hospital, Munich, Germany; 6 Medical Physics, Department of Radiology, Medical Centre - University of Freiburg, Freiburg, Germany; 7 Institute of Biometrics and Epidemiology, German Diabetes Center, Duesseldorf, Germany; 8 German Center for Diabetes Research (DZD), Partner Site Neuherberg, Neuherberg, Germany; 9 Department of Neuroradiology, Medical Center - University of Freiburg, Faculty of Medicine, University of Freiburg, Breisacher Str. 64, 79106 Freiburg, Germany

**Keywords:** Biomarkers, Cardiology, Endocrinology, Medical research, Pathogenesis, Risk factors

## Abstract

Aim of this study was to analyse the associations of cardiovascular health and adrenal gland volume as a rather new imaging biomarker of chronic hypothalamic–pituitary–adrenal (HPA) axis activation. The study population originates from the KORA population-based cross-sectional prospective cohort. 400 participants without known cardiovascular disease underwent a whole-body MRI. Manual segmentation of adrenal glands was performed on VIBE-Dixon gradient-echo sequence. MRI based evaluation of cardiac parameters was achieved semi-automatically. Cardiometabolic risk factors were obtained through standardized interviews and medical examination. Univariate and multivariate associations were derived. Bi-directional causal mediation analysis was performed. 351 participants were eligible for analysis (56 ± 9.1 years, male 58.7%). In multivariate analysis, significant associations were observed between adrenal gland volume and hypertension (outcome hypertension: Odds Ratio = 1.11, 95% CI [1.01, 1.21], p = 0.028), left ventricular remodelling index (LVRI) (outcome LVRI: β = 0.01, 95% CI [0.00, 0.02], p = 0.011), and left ventricular (LV) wall thickness (outcome LV wall thickness: β = 0.06, 95% CI [0.02, 0.09], p = 0.005). In bi-directional causal mediation analysis adrenal gland volume had a borderline significant mediating effect on the association between hypertension and LVRI (p = 0.052) as well as wall thickness (p = 0.054). MRI-based assessment of adrenal gland enlargement is associated with hypertension and LV remodelling. Adrenal gland volume may serve as an indirect cardiovascular imaging biomarker.

## Introduction

External and internal stressors, including environmental, physiological and psychological factors lead to the activation of the stress response, which involves the hypothalamic–pituitary–adrenal (HPA) axis^1–3^. While acute and transient HPA axis activation has an adaptive function^4^, prolonged and repeated cortisol exposure may provoke long-term physiological alterations leading to pathological conditions affecting the cardiovascular, metabolic, immune and nervous systems^5^.

Cardiovascular disease (CVD) is a leading contributor to the burden of morbidity and mortality worldwide^6^. Various cardiometabolic risk factors have been identified, including high blood pressure, diabetes, obesity, and elevated cholesterol levels^7^. Additionally to these well-established cardiometabolic risk factors, elevated cortisol levels seem to be associated with cardiovascular risk factors and cardiovascular diseases^8,9^. For example, dysregulation of the HPA axis is thought to be involved in the pathogenesis of high blood pressure^10^ and clinical syndromes involving chronic elevations of cortisol levels (such as Cushing’s disease) are frequently associated with hypertension^11^. Furthermore, previous findings support the development of interventions that effectively lower cortisol to reduce the risk of CVD^7^. These findings indicate that dysregulation of the HPA axis contributes to cardiovascular and metabolic alterations, entangled in complex pathomechanisms. Whether cardiovascular health risks arise from direct damaging effects of stress activation or whether pathologies develop secondary to the accompanying metabolic strain of excess glucocorticoids remains unclear^12^. Hence, research on HPA axis activation as a potentially modifiable risk factor is of major public health concern.

Magnetic resonance imaging (MRI) provides the opportunity to assess and visualize cardiovascular and metabolic parameters^13^. Thus, cardiac MRI enables the assessment of structural and functional cardiac alterations such as end-diastolic or end-systolic volume and left ventricular mass (LVM). LVM is recognized as a risk factor for cardiovascular events^14^, and furthermore, the left ventricular remodelling index (LVRI)—defined as a modification in shape, size, and function of the left ventricle due to physiological or pathological conditions—represents a simple parameter for the investigation of cardiac adaptations to physiological and pathological conditions^15,16^. In addition, we recently showed that MRI is an appropriate non-invasive modality for the assessment of adrenal gland volume^17^, which can be considered as an indirect imaging biomarker for chronic activation of the HPA axis, as chronic activation of the HPA axis increases the volume of adrenal glands through trophic effects of adrenocorticotropic hormone (ACTH)^18^, which is considered the predominant if not the exclusive trophic factor for the adrenals^19^. Inversely, it has been reported that hypophysectomy results in adrenal cortex atrophy that is restored by the sole administration of ACTH^20^.

Our study aimed to analyse the associations of cardiovascular health and adrenal gland volume assessed by MRI as an indirect imaging biomarker of chronic HPA axis activation in a general population without prior known CVD.

Our hypothesis is that the HPA axis is directly involved in the occurrence of established cardiovascular risk factors, and an increased adrenal gland volume may reveal itself as a possible indirect imaging biomarker of CVD, even in subclinical disease.

## Results

400 participants were enrolled in the KORA-MRI sub-study. After exclusion of participants with missing or incomplete T1w VIBE-Dixon sequences (n = 25), missing or bad image quality of cine-steady state free precession sequences (n = 22), detection of adrenal gland incidentalomas (n = 2), and with regular systemic corticoid intake (n = 2), a total of 351 participants was eligible for the analysis of associations between adrenal gland volume and cardiac alterations. Data on LV wall-thickness were missing for 2 further participants.

The mean age of the participants was 56 ± 9.1 years (male 58.7%). The average sum of right and left adrenal gland volume (total adrenal gland volume) was 11.1 ± 4.2 ml. Hypertension was present in 33%. Obesity was detected in 29.1% and overweight in 43.6%. 92 (26.2%) of the participants had prediabetes and 44 (12.5%) had T2DM (Table 1).

**Table 1:** Participant’s demographic, clinical and cardiovascular parameters.

	All
N	351
Age (years)	56.0 ± 9.1
Male sex	206 (58.7%)
Female sex	145 (41.3%)
Adrenal gland volume	
Adrenal gland volume, ml (right)	5.2 ± 2.1
Adrenal gland volume, ml (left)	5.9 ± 2.4
Adrenal gland volume, ml (sum of right and left)	11.1 ± 4.2
Adrenal gland volume, ml (average of left and right)	5.5 ± 2.1
Anthropometrics	
Height (cm)	171.9 ± 9.6
BMI (kg/m^2^)	28.0 ± 4.7
Normal weight (< 25 kg/m^2^)	96 (27.4%)
Overweight (25–29.9 kg/m^2^)	153 (43.6%)
Obese (≥ 30 kg/m^2^)	102 (29.1%)
Lipid metabolism	
Triglycerides (mg/dl)	132.3 ± 86.0
Total cholesterol (mg/dl)	217.3 ± 36.5
HDL (mg/dl)	61.6 ± 17.2
LDL (mg/dl)	139.3 ± 33.1
Lipid-lowering medication	35 (10.0%)
Blood pressure	
Systolic blood pressure (mmHg)	120.5 ± 16.7
Diastolic blood pressure (mmHg)	75.5 ± 9.9
Hypertension	116 (33.0%)
Antihypertensive medication	84 (23.9%)
Cardiac function	
LV end-diastolic volume (ml/m^2^)	66.4 ± 15.1
LV end-systolic volume (ml/m^2^)	20.9 ± 8.7
LV ejection fraction (%)	69.2 ± 7.8
LVM (g/m^2^)	71.7 ± 13.9
LVRI	1.1 ± 0.3
Mean LV end-diastolic wall thickness (mm) (N = 349)	9.5 ± 1.5
Glucose metabolism	
Glycemic status	
Normoglycemia	215 (61.3%)
Prediabetes	92 (26.2%)
T2DM	44 (12.5%)
2 h Glucose (mg/dl) / oGTT	113.1 ± 40.1
Fasting glucose (mg/dl)	104.2 ± 22.9
HbA1c (%)	5.6 ± 0.7
Antidiabetic medication	26 (7.4%)
Behavioral risk factors	
Physically active	209 (59.5%)
Alcohol consumption (g/d) Median [IQR]	10.1 [1.4, 27.5]
No	79 (22.5%)
Moderate	202 (57.5%)
Heavy	70 (19.9%)
Smoking	
Never-smoker	120 (34.2%)
Ex-smoker	160 (45.6%)
Smoker	71 (20.2%)

For continuous variables, values are mean ± standard deviation. For categorical variables, values are counts and percentages.

*T2DM* type 2 diabetes mellitus, *BMI* body mass index, *HDL* high density lipoprotein, *LDL* low density lipoprotein, *LV* left ventricular, *LVM* left ventricular myocardial mass, *LVRI* left ventricular remodelling index, *oGTT* oral glucose tolerance test.

### Adrenal gland volume and hypertension

**Figure 1:**
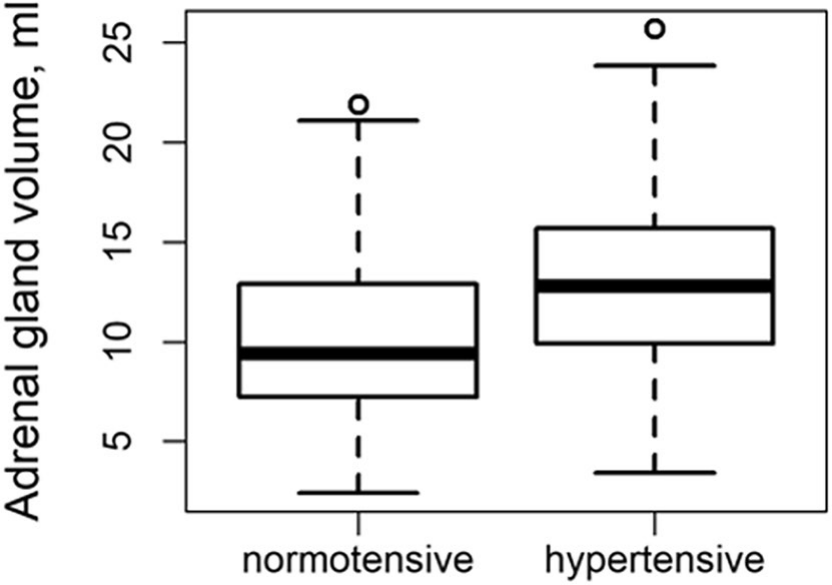
Distribution of adrenal gland volume according to presence of hypertension.

An association between adrenal gland volume and hypertension was observed, which remained statistically significant after adjustment for metabolic confounders (outcome hypertension: OR_AdrenalGlandVolume_ = 1.10, 95% CI [1.01, 1.21], p = 0.030). Analogously, systolic blood pressure remained significantly associated with adrenal gland volume after stepwise adjustment (outcome systolic blood pressure: β_AdrenalGlandVolume_ = 0.57, 95% CI [0.06, 1.07], p = 0.029), while the association of diastolic blood pressure and adrenal gland volume was attenuated after adjustment for lipometabolic parameters. (Table 2, Fig. 1).

**Table 2:** Association of adrenal gland volume with outcome cardiovascular parameters in multivariable analysis.

	Predictor adrenal gland volume (ml)		
	Beta/OR	95% CI	p-value
Adjusted for age and sex			
Outcome			
LVRI	0.02	[0.02, 0.03]	< 0.001
LVM (g/m^2^)	0.53	[0.17, 0.89]	0.004
LV end-diastolic volume (ml/m^2^)	− 0.77	[− 1.20, − 0.34]	< 0.001
LV wall thickness (mm)	0.14	[0.10, 0.17]	< 0.001
Hypertension	1.20	[1.12, 1.29]	< 0.001
Systolic blood pressure (mmHg)	1.17	[0.75, 1.59]	< 0.001
Diastolic blood pressure (mmHg)	0.70	[0.43, 0.97]	< 0.001
Adjusted for age, sex, BMI, triglycerides, LDL and total cholesterol			
Outcome			
LVRI	0.02	[0.01, 0.03]	< 0.001
LVM (g/m^2^)	0.48	[0.05, 0.92]	0.031
LV end-diastolic volume (ml/m^2^)	− 0.32	[− 0.83, 0.20]	0.226
LV wall thickness (mm)	0.08	[0.04, 0.12]	< 0.001
Hypertension	1.12	[1.03, 1.22]	0.006
Systolic blood pressure (mmHg)	0.64	[0.14, 1.14]	0.013
Diastolic blood pressure (mmHg)	0.29	[− 0.03, 0.61]	0.078
Adjusted for age, sex, BMI, triglycerides, LDL, total cholesterol, diabetes status, alcohol consumption and smoking habits			
Outcome			
LVRI	0.01	[0.00, 0.02]	0.003
LVM (g/m^2^)	0.47	[0.02, 0.91]	0.040
LV end-diastolic volume (ml/m^2^)	− 0.18	[− 0.68, 0.33]	0.498
LV wall thickness (mm)	0.07	[0.03, 0.11]	< 0.001
Hypertension	1.11	[1.01, 1.21]	0.028
Systolic blood pressure (mmHg)	0.57	[0.06, 1.08]	0.029
Diastolic blood pressure (mmHg)	0.28	[− 0.05, 0.61]	0.092
Adjusted for age, sex, BMI, triglycerides, LDL, total cholesterol, diabetes status, alcohol consumption, smoking habits and systolic blood pressure			
Outcome			
LVRI	0.01	[0.00, 0.02]	0.011
LVM (g/m^2^)	0.31	[− 0.11, 0.74]	0.150
LV end-diastolic volume (ml/m^2^)	− 0.17	[− 0.68, 0.34]	0.508
LV wall thickness (mm)	0.06	[0.02, 0.09]	0.005

Adrenal gland volume is not standardized. All estimates are given as Beta-coefficients from linear regression, except for hypertension, where estimates are given as Odds Ratios from logistic regression.

*CI* confidence interval, *LVRI* left ventricular remodelling index, *LVM* left ventricular myocardial mass, *LV* left ventricular, *BMI* body mass index, *LDL* low density lipoprotein.

### Adrenal gland volume and LV remodelling

**Figure 2:**
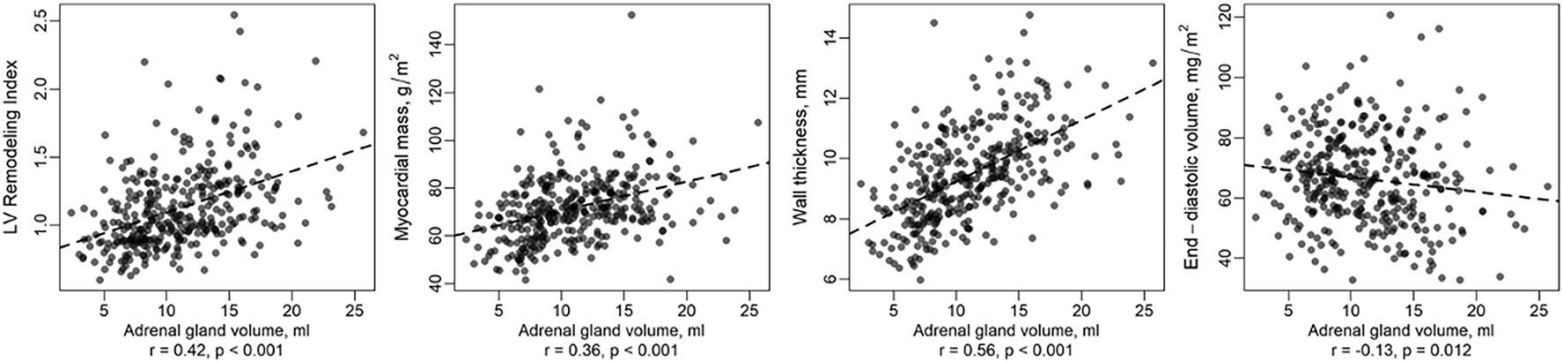
Correlation between adrenal gland volume and LVRI/LVM/left ventricular wall thickness/end-diastolic volume.

LVRI and adrenal gland volume showed a consistent association after extensive adjustment (outcome LVRI: β_AdrenalGlandVolume_ = 0.01, 95% CI [0.00, 0.02], p = 0.011). And stable association between LV wall thickness and adrenal gland volume was observed (after adjustment for age, sex, BMI, lipometabolic parameters, diabetes status, alcohol consumption, smoking habits and systolic blood pressure: outcome LV wall thickness: β_AdrenalGlandVOlume_ = 0.06, 95% CI [0.02, 0.09], p = 0.005) (Table 2, Fig. 2).

### Adrenal gland volume and cardiac function parameters

Initially, the cardiac function parameter LV end-diastolic volume indicated a connection with adrenal gland volume (adjusted for age and sex: outcome LV end-diastolic volume: β = − 0.77, 95% CI [− 1.20, − 0.34], p < 0.001), but after further stepwise adjustment in linear regression analysis the association became non-significant.

Similarly, the cardiac function parameter LVM initially indicated a connection with adrenal gland volume (adjusted for age and sex, BMI, lipometabolic parameters, diabetes status, alcohol consumption and smoking habits: outcome LVM: β = 0.47, 95% CI [0.02, 0.91], p = 0.04), but after further adjustment for systolic blood pressure the association became non-significant (Table 2).

### Mediating effects in the association of adrenal gland volume, hypertension and cardiac remodelling

**Figure 3:**
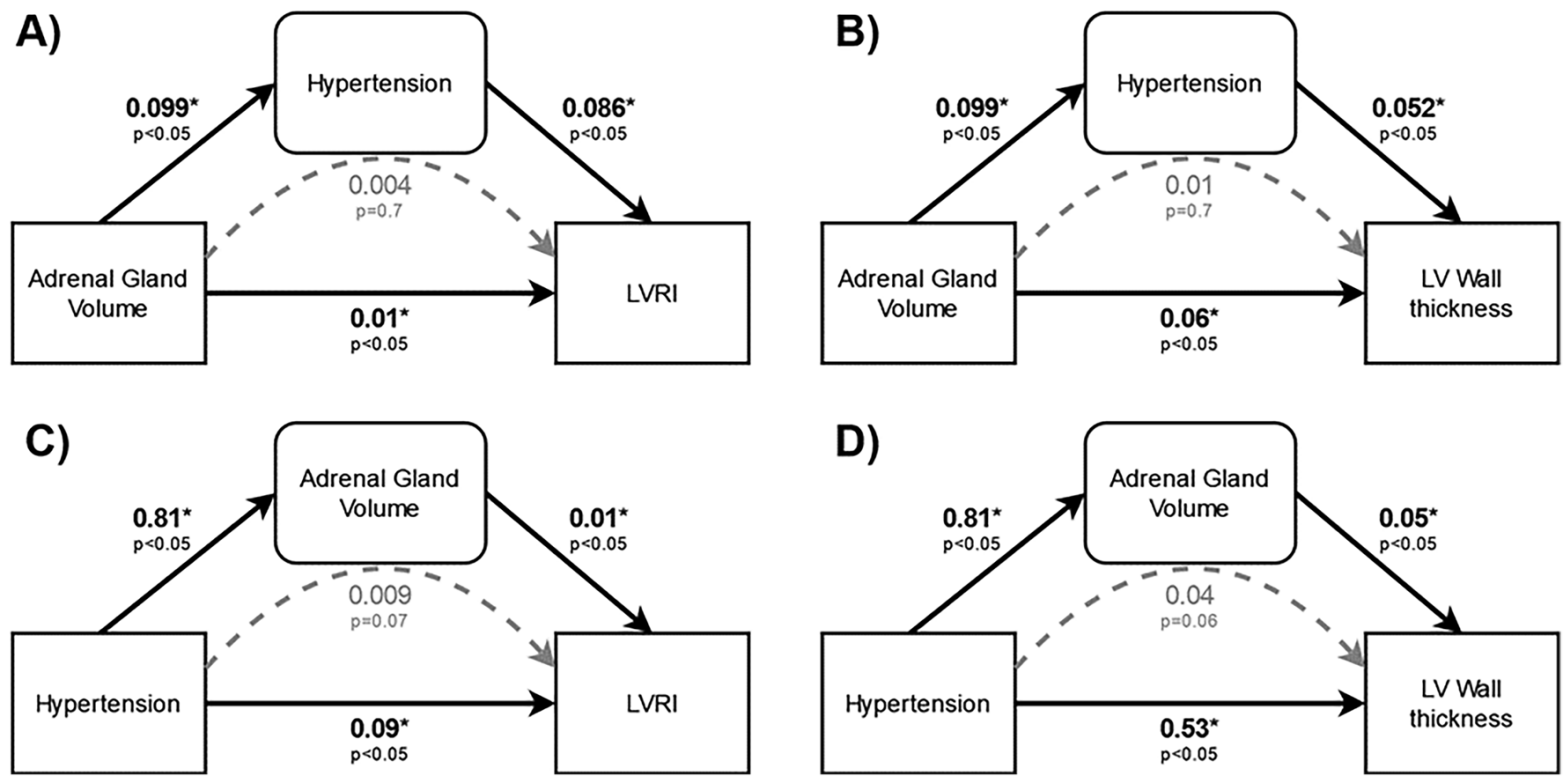
Causal mediation analysis on the relationship between adrenal gland volume, hypertension and cardiac remodelling. Upper panels (**A**) and (**B**) show exposure adrenal gland volume with mediator hypertension, Lower panels (**C**) and (**D**) show exposure hypertension with mediator adrenal gland volume. Direct effects are indicated by solid black arrows whereas mediation effects are indicated by dashed grey arrows. Labels indicate effect size and p-values.

Using LVRI and LV wall thickness as outcome variables, we performed causal mediation analysis with adrenal gland volume and hypertension as exposure and mediation variables. Hypertension did not mediate the effect of adrenal gland volume on either LVRI (p = 0.7) or LV wall thickness (p = 0.7) (Fig. 3A,B). On the other hand, adrenal gland volume had a borderline significant mediating effect on the association between hypertension and LVRI (p = 0.052) and LV wall thickness (p = 0.054) (Fig. 3C,D). Conceptually, this would indicate that a part of the risk for cardiac remodelling conferred by hypertension is due to the effect of hypertension on adrenal gland enlargement.

## Discussion

This study investigated the relationship between adrenal gland volume and cardiovascular alterations, assessed by MRI in subjects without prior CVD. We found strong associations between enlargement of adrenal gland volume and hypertension, LVRI as well as LV wall thickness that persisted after extensive adjustment for possible confounders. Furthermore, we found tentative evidence that the effect of hypertension on cardiac remodelling might partly be channelled through adrenal gland volume. Overall, our findings support the assumption that increased volume of the adrenal glands is associated with cardiovascular alterations in the form of LV remodelling and hypertension, independent of other traditional cardiovascular risk factors, and thus may serve as an indirect imaging biomarker for dysregulated HPA axis even in a subclinical state.

Conventionally, HPA axis activity is assessed through cortisol levels in blood, urine and saliva^21^, limiting analyses to momentary or short-term estimates of cortisol levels^22^. However, prolonged and repeated exposure to HPA axis activation likely plays a much more important role in the development of pathologies than acute or transient cortisol exposure^23^. Recently, the quantification of cortisol in hair has been introduced as a novel biomarker of chronic cortisol exposure^8^. But, hair samples may not be easy to obtain in retrospective analysis settings. Previously, we have shown that MRI is an appropriate non-invasive modality for assessing adrenal gland volume^17^. Chronic activation of the HPA axis increases the volume of adrenal glands through trophic effects of ACTH and is considered the predominant if not the exclusive trophic factor for the adrenals^18,19^. Thus, we propose adrenal gland enlargement as an indirect imaging biomarker of chronic cortisol exposure.

Dysregulation of the HPA axis is thought to be involved in the pathogenesis of high blood pressure^11^. In line with this assumption, our study showed a strong correlation between adrenal gland volume and hypertension, which interestingly was independent of possible confounders associated with the metabolic syndrome, suggesting a direct effect of hypertension and HPA axis activity on each other. Although elevated cortisol and HPA axis dysregulations have already been shown to be associated with increased blood pressure^11^, the links between HPA axis dysregulation, adrenal gland enlargement and hypertension are not quite clear.

Pathological changes of LVRI can be seen in different primary and secondary disorders of the ventricles such as ischemic cardiomyopathy or hypertension^24,25^.

Storz et al. previously showed that in the KORA-MRI study participants with prediabetes and diabetes without prior cardiovascular disease had significantly higher LVRI than healthy controls, independent of BMI, hypertension, age and sex^26^. These results probably mirror the risk of development of diabetic cardiomyopathy in case of underlying impaired glucose metabolism^27^. On the other side, our results demonstrate an association between adrenal gland enlargement and LVRI independently from diabetes status, hypertension, and further possible cardiometabolic confounders, and highlight the extent of consequences of chronic stress for subclinical cardiac alterations in study participants without apparent cardiovascular disease.

Interestingly, our results did not reveal a significant persisting correlation between LVM or LV end-diastolic volume and adrenal gland volume, since associations became non-significant for both parameters after adjusting for confounding variables. In contrast, LV wall thickness remained significantly associated with adrenal gland volume after extensive adjustment for possible cardiometabolic confounders.

To disentangle the complex interplays between the HPA axis and cardiovascular alterations, we performed a bi-directional causal mediation analysis, which showed that adrenal gland volume rather than hypertension had a borderline mediating effect on the association between hypertension and LVRI as well as LV wall thickness.

It is well known that patients with hypertensive heart disease generally develop LV remodelling^25^, and hypertension is one of the most common causes of LV hypertrophy^28^. However, our results show that the impact of hypertension on cardiac remodelling might be partially mediated through adrenal gland volume.

Limitations of our study have to be reflected. Our study cohort consisted of a population without manifest cardiovascular diseases from the region of Augsburg, diminishing our results’ generalizability to other ethnicities, geographical regions or patients with cardiovascular diseases. Furthermore, the participants’ cortisol levels were not collected, which would have strengthened our observations on HPA axis activation. Also, MRI-based quantification of adrenal gland volume as a marker for chronic HPA axis activation is a newer imaging biomarker, which still has to establish itself as a standard method for epidemiological studies. Still, we previously demonstrated the feasibility and appropriateness of the non-invasive modality^17^, and the connection between constant HPA axis activation and enlargement of adrenal glands has previously been discussed^18–20^. Our study has a cross-sectional design and therefore can neither demonstrate the chronology of events and the observed relationships nor allows final conclusions on the development of hypertension or LV remodelling depending on HPA axis dysregulation. However, the investigated study cohort consisted of participants without known or manifest cardiovascular disease, suggesting an early involvement of HPA axis activation in subclinical cardiovascular alterations. Furthermore, we performed causal mediation analysis through which we found a potential mediating effect of adrenal gland volume on the association between hypertension and cardiac remodelling.

Further limitations of the present study are missing data on 24-h ambulatory blood pressure measurement. Dexamethasone suppression tests or other tests to exclude hypercortisolism or autonomous cortisol excretion were not performed and data on Cushing’s syndrome, primary aldosteronism, pheochromocytoma were missing. Although prevalence of these diseases is rather low, our findings should not be translated to individuals with these disorders. In addition, participants with adrenal gland incidentalomas were excluded from the data analysis.

Catecholamine secretion was also not measured in this study, but as ACTH is considered the predominant if not the exclusive trophic factor for the adrenals^19^, we presume that these missing data would also not alter our results. Nevertheless, it has to be conceded that missing information on possible confounders regarding adrenal gland volume may impact the reliability of our results. In particular, analysis of cortisol levels and its association with adrenal gland volume will provide additional insights that will shed more light on their respective roles in HPA axis activation.

Further studies should elucidate in a longitudinal study design, if and how HPA axis activation may trigger cardiac alterations, whether these may develop into manifest cardiac diseases, and—if applicable—determine cut-off values of adrenal gland volume for the development of cardiovascular diseases.

## Conclusions

In this study, we found strong correlations between adrenal gland volume and hypertension, LVRI as well as LV wall thickness independent of established cardiovascular risk factors. Furthermore, through causal mediation analysis, we found tentative statistical evidence that the effect of hypertension on cardiac remodelling might partly be channelled through HPA axis activation as defined by adrenal gland enlargement. Thus, MRI-based assessment of adrenal gland volume represents an indirect imaging marker for cardiovascular burden even in subjects without prior CVD.

Assuming, that hypertension and cardiac remodelling, in the form of increased LVRI and increased LV wall thickness, represent cardiometabolic risk factors and potential disruptive factors affecting the HPA axis, the associated enlargement of adrenal glands assessed by MRI may represent a further imaging marker for cardiovascular burden even in subjects without prior CVD. However, due to the cross-sectional study design and missing cortisol levels our study results do not permit final conclusions on the development of hypertension or LV remodelling depending on HPA axis dysregulation.

Further studies are needed to elucidate, if and how adrenal gland enlargement or HPA axis dysregulation is involved in the development of manifest CVD and whether interventions targeting hypercortisolism may reduce the development or presence of cardiovascular and metabolic alterations and thus may prevent cardiovascular events.

## Materials and methods

### Study design and study population

The study sample consists of a cross-sectional imaging sub-study (KORA-MRI) of a population-based prospective cohort from the ‘Cooperative Health Research in the Augsburg Region, Germany’ (KORA-FF4), which investigated the interplays between subclinical metabolic and cardiovascular disease in individuals with impaired glucose metabolism^13,29^. The KORA-MRI sub-study included 400 participants, who underwent a whole-body MRI study protocol between June 2013 and September 2014. Exclusion criteria consisted of prior cardiovascular disease (based on physician-validated self-report: stroke, myocardial infarction, or any revascularization, cardiac pacemaker or implantable defibrillator), contraindications to MRI and/or gadolinium contrast agent, and age > 72 years, as described previously^13^. Further exclusion criteria for the present study were the detection of adrenal gland incidentalomas, poor image quality for the assessment of adrenal gland volume, missing data sets or poor image quality for the assessment of cardiac imaging parameters, and a systemic cortisol intake.

### Ethics declaration

The Institutional Research Ethics Board of the Medical Faculty of Ludwig-Maximilian University Munich approved the KORA-MRI study, the requirements of the Helsinki declaration on human research were met, and all participants gave their written informed consent.

### MR-imaging protocol

The MRI study protocol included various sequences for the chest and abdomen, as well as for the cardiovascular system, as detailed elsewhere^13^.

### MR-image analysis

#### Adrenal gland volume

**Figure 4:**
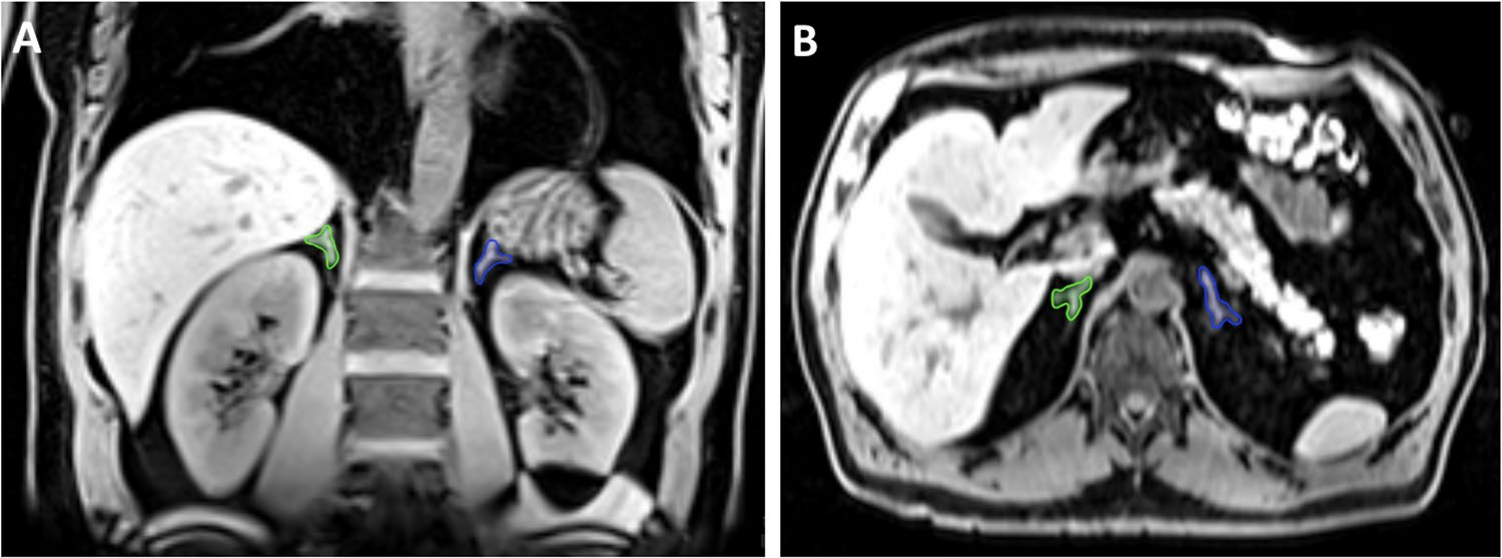
Example of adrenal gland segmentation of the right adrenal gland (green) and of the left adrenal gland (blue) in coronal (**A**) and axial (**B**) reconstructions on T1w-VIBE-Dixon sequences.

Bilateral segmentation of adrenal gland volume was achieved through manual segmentation of the organ on the water-only phase sequences of the two-point T1 weighted isotropic VIBE-Dixon gradient-echo sequence with the medical imaging platform NORA (http://www.nora-imaging.com), and the volume was calculated as the number of voxels multiplied by voxel size. Previously, we have shown that this procedure is an appropriate non‐invasive modality for the assessment of adrenal gland volume with appropriate intra‐ and interrater reliability^17^. The main parameter of interest for the current study was total adrenal gland volume, defined as the sum of left and right. Figure 4 provides an example of adrenal gland segmentation in coronal and axial reconstructions.

#### Cardiac parameters

**Figure 5:**
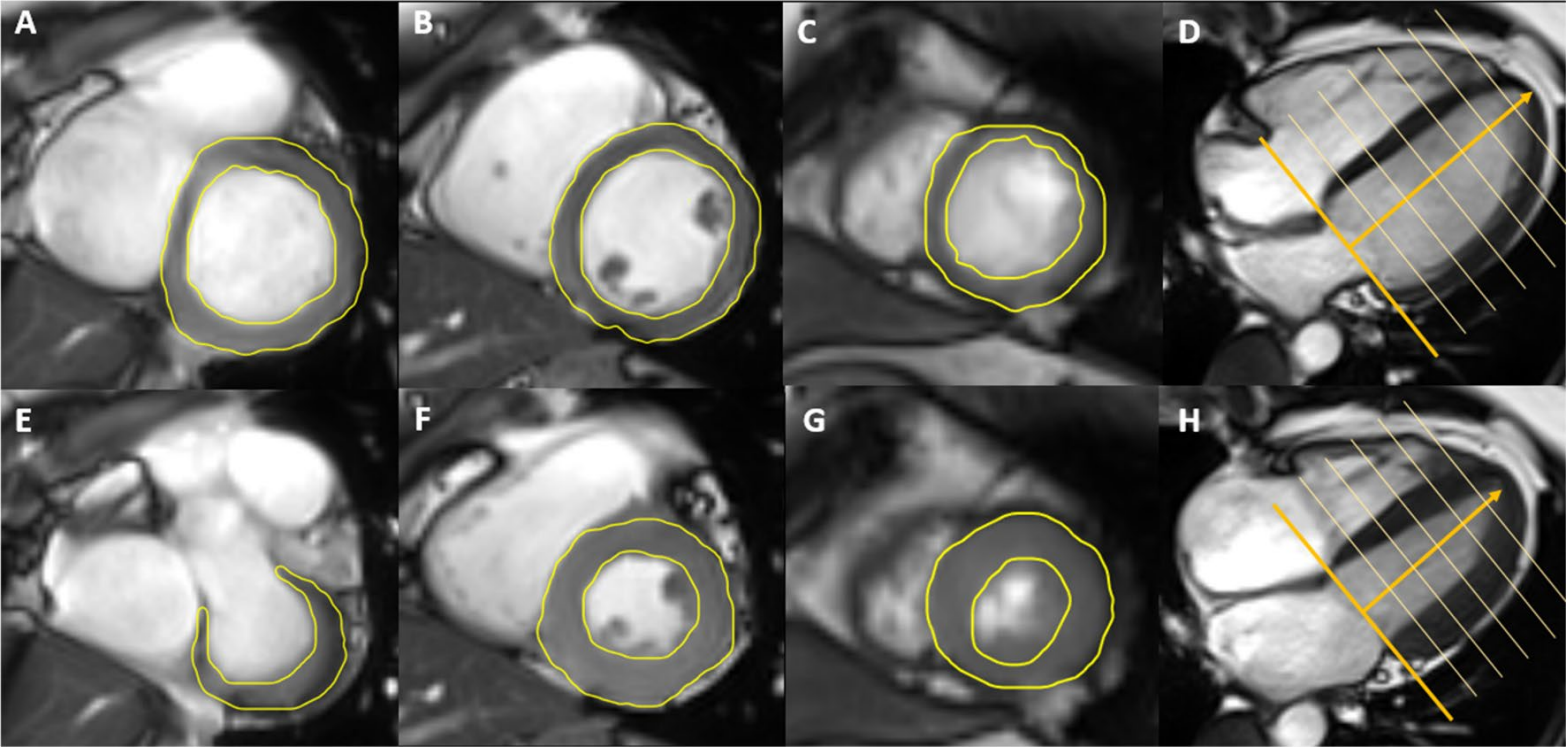
Example of left ventricular segmentation on cine-steady state free precession sequences to derive left ventricular volume and functional parameters. Basal diastolic (**A**), mid-ventricular diastolic (**B**) and apical diastolic (**C**) short axis images and diastolic 4-chamber-view (**D**). Basal systolic (**E**), mid-ventricular systolic (**F**) and apical systolic (**G**) short axis images and systolic 4-chamber-view (**H**). The outer yellow line indicates the epicardial contour, the inner yellow line indicates the endocardial contour (**A**–**C**,**E**,**F**). The orange lines indicate the tagging of the left ventricle on the 4-chamber view (**D**,**H**).

The cardiac parameters analysed in this study relate to the left ventricle. The evaluation of cardiac parameters took place by semi-automatic contouring of the endo- and epicardium on cine-steady state free precession sequences acquired in four chamber view and short axis stack with 10 slices and 25 phases with a dedicated software package (cvi42, Version 4.1.5(190), Circle Cardiovascular Imaging Inc., Calgary, Canada). Thereof, the following cardiac parameters were derived: left-ventricular (LV) end-systolic volume, LV end-diastolic volume, LV ejection fraction, LVM (end-diastolic) and LV wall thickness (end-diastolic)^13^, since these are established clinical markers of cardiac morphology and function, which have established themselves as imaging biomarkers for CVD^30^, and MRI has shown a high degree of reproducibility for these parameters^31^. The LVRI was calculated as the ratio of LVM to LV end-diastolic volume^26^. Volumes and mass were indexed to body surface area, as calculated by duBois formula. Missing values in MRI parameters were due to incomplete protocol acquisition, technical artefacts or low image quality and independent of participants’ characteristics. Figure 5 provides an example of left ventricular segmentation on cine-steady state free precession sequences.

### Other covariates

Sociodemographic data, cardiometabolic risk factors, and health-related covariates were collected through standardized interviews, medical examination, anthropometric measurements, and blood sampling^13^. Systolic and diastolic blood pressure was measured on the right arm in seated subjects after 5 min resting and was repeated three times. The average of the last two acquired values was taken for statistical evaluation. Hypertension was defined as increased systolic blood pressure ≥ 140 mmHg, increased diastolic blood pressure ≥ 90 mmHg or/and intake of antihypertensive medication^13^. For assessment of diabetes status, normal fasting glucose concentration (FPG), 2-h serum glucose concentration from oral glucose tolerance testing (oGTT), and use of antidiabetic medication were evaluated, and the 1998 World Health Organization criteria were applied to define prediabetes and type 2 diabetes (T2DM)^32^. Body mass index (BMI) was defined as the value from the participant in kilograms divided by their height squared in meters, and the categorization into normal, overweight, and obese was made according to the definition of the world health organization (normal BMI < 25 kg/m^2^, overweight BMI 25 to < 30 kg/m^2^, obese BMI ≥ 30 kg/m^2^)^33^. Alcohol consumption was assessed in g/d and was classified into three categories: no alcohol consumption (0 g/d), moderate alcohol consumption (0.1–39.9 g/d for men and 0.1–19.9 g/d for women), and heavy alcohol consumption (≥ 40 g/d for men and ≥ 20 g/d for women)^34^. Smoking habits were categorized into ‘never-smoker’, ‘ex-smoker’ and ‘smoker’, based on self-report.

### Statistical analysis

Participants’ characteristics are described as mean and standard deviation and counts and percentages for continuous and categorical variables, respectively. Distributions of continuous data were visually inspected for adequate normality to perform parametric testing. Correlations between adrenal gland volume and cardiovascular risk factors are visualized by scatter plots and quantified by Pearson’s correlation coefficient. To assess the association between adrenal gland volume and markers of subclinical cardiovascular disease, multiple regression models with stepwise adjustment were calculated.

Linear regression models were calculated for outcome cardiovascular parameters with stepwise adjustment for (1) age and sex, (2) age, sex, BMI, triglycerides, LDL and total cholesterol, (3) as in (2) plus diabetes status, alcohol consumption and smoking habits, (4) as in (3) plus systolic blood pressure. Adrenal gland volume served as exposure of interest. Before analysis, all continuous variables were standardized (minus mean and divided by standard deviation). Results from linear regression models are reported as β-coefficients with corresponding 95%-confidence intervals (CI), indicating a change in the outcome per standard deviation change of the exposure. Model diagnostics were performed to ensure adequate adherence to assumptions for linear regression. Analogously, results from logistic regression are reported as Odds Ratios.

To further characterize the relationship between adrenal gland volume, hypertension, and cardiac morphology, we performed bi-directional causal mediation analysis, which provides a statistical framework for a hypothesized pathway of associations.

We used either hypertension or adrenal gland volume as the exposure and the respective other variable as a potential intermediary factor on the causal pathway.

P-values < 0.05 were considered to indicate statistical significance. All analyses were performed with R version 4.1.2^35^.

### Ethics approval and consent to participate

The Institutional Research Ethics Board of the Medical Faculty of Ludwig-Maximilian University Munich approved the KORA-MRI study, the requirements of the Helsinki declaration on human research were met, and all participants gave their written informed consent.

## Data Availability

Datasets generated during and/or analyzed during the current study are not publicly available but are available from the corresponding author on reasonable request.

## Abbreviations

ACTH: Adrenocorticotropic hormone
BMI: Body mass index
CI: Confidence interval
CVD: Cardiovascular disease
FPG: Fasting plasma glucose
HPA: Hypothalamic–pituitary–adrenal (axis)
KORA: Cooperative Health Research in the Augsburg Region, Germany
LDL: Low density lipoprotein
LV: Left ventricular
LVM: Left ventricular mass
LVRI: Left ventricular remodelling index
MRI: Magnetic resonance imaging
oGTT: Oral glucose tolerance test
OR: Odds ratio
SD: Standard deviation
T2DM: Type 2 diabetes mellitus
